# Modeling AP2M1 developmental and epileptic encephalopathy in *Drosophila*

**DOI:** 10.1242/dmm.052419

**Published:** 2025-11-21

**Authors:** Robin A. Karge, Florian P. Fischer, Hannah Schüth, Aileen Wechner, Sabrina Peter, Lukas A. Kilo, Mato Dichter, Aaron Voigt, Gaia Tavosanis, Karen M. J. van Loo, Henner Koch, Yvonne G. Weber, Stefan Wolking

**Affiliations:** ^1^ Section of Epileptology, Department of Neurology, RWTH Aachen University, 52074 Aachen, Germany; ^2^ Department of Developmental Biology, Institute for Neurobiology and Biomedical Life Science, RWTH Aachen University, 52074 Aachen, Germany; ^3^ Department of Neurology, RWTH Aachen University, 52074 Aachen, Germany; ^4^ JARA-BRAIN Institute Molecular Neuroscience and Neuroimaging, Forschungszentrum Jülich GmbH and RWTH Aachen University, 52074 Aachen, Germany

**Keywords:** *Drosophila melanogaster*, Epilepsy, Genetics, Variant modeling, *AP2M1*

## Abstract

Genetic defects in *AP2M1*, which encodes the μ-subunit of the adaptor protein complex 2 (AP-2) essential for clathrin-mediated endocytosis, cause a rare form of developmental and epileptic encephalopathy (DEE). In this study, we modeled *AP2M1*-DEE in *Drosophila melanogaster* to gain deeper insights into the underlying disease mechanisms. Pan-neuronal RNA interference against the *Drosophila AP2M1* ortholog, *AP-2µ*, resulted in a consistent heat-sensitive paralysis phenotype and altered morphology in class IV dendritic arborization neurons. Unexpectedly, affected flies were resistant to antiseizure medications and exhibited decreased susceptibility to electrically induced seizures. A CRISPR-engineered fly line carrying the recurrent human disease variant p.Arg170Trp displayed a milder, seizure-resistant phenotype. Although these findings contrast with the human phenotype, they align with previous studies on other clathrin-mediated endocytosis-related genes in *Drosophila*. Our results suggest that hyperexcitability and seizures in *AP2M1*-DEE may stem from broader defects in neuronal development rather than direct synaptic dysfunction.

## Teaser

**Summary:** Dysfunction of the *Drosophila* endocytic adaptor gene *AP-2µ* leads to decreased seizure susceptibility and resistance to antiseizure medications, suggesting that hyperexcitability and seizures in human *AP2M1* developmental and epileptic encephalopathy are not caused by direct synaptic dysfunction.

## INTRODUCTION

Epilepsies are defined by recurring epileptic seizures and rank among the most prevalent neurological disorders (Ngugi et al., 2010). Genetic causes have been identified for at least 20% of all people with epilepsy, encompassing polygenic syndromes such as idiopathic generalized epilepsies and focal epilepsies (ILAE, 2023; May et al., 2018; Epi25, 2024). Although rare, monogenic epilepsies, particularly in the form of developmental and epileptic encephalopathies (DEEs), have a significant clinical and socioeconomic impact (Salcedo-Perez-Juana et al., 2023). DEEs manifest in newborns or early childhood, characterized by commonly intractable seizures, intellectual impairment and lifelong disability (Wolking and Weber, 2015).

In the gene *AP2M1*, the recurrent *de novo* variant c.508C>T (p.Arg170Trp) (GenBank: NM_004068.3) has been detected in four individuals with DEE, featuring a DEE subtype of epilepsy with myoclonic-atonic seizures (Helbig et al., 2019). *AP2M1* encodes the µ-subunit of the adaptor protein complex 2 (AP-2). *AP2M1* is highly intolerant to genetic variation. Gene constraint analyses (Lek et al., 2016; Karczewski et al., 2020) show a probability of loss-of-function intolerance (pLI) score of 1, a low observed/expected ratio (0.09) for loss-of-function variants, and a high missense Z-score (Z=6.37). These findings indicate that loss-of-function variants in *AP2M1* are exceedingly rare (Helbig et al., 2019). Knockout studies in mice have underscored the essential role of *Ap2m1* in embryonic development. Complete gene inactivation results in embryonic lethality as early as embryonic day (E)3.5 (Mitsunari et al., 2005). However, heterozygous knockout mice display no overt phenotype, suggesting that a single functional copy of *Ap2m1* is sufficient for normal development. Conditional knockout of *Ap2m1* in neurons resulted in reduced neuronal complexity and impaired neuronal viability, emphasizing its crucial role in neuronal development and survival (Kononenko et al., 2017).

At the molecular level, *AP2M1* is a key component of clathrin-mediated endocytosis (CME) of membrane proteins (Azarnia Tehran et al., 2019). CME is a fundamental cellular process for the internalization of essential molecules, including nutrients, vesicle proteins, hormones and receptors, through vesicle formation at the plasma membrane. Central to this process is the adaptor protein complex 2, a heterotetramer composed of α, β2, σ2 and µ2 subunits (Mitsunari et al., 2005). AP-2 acts as a central hub, coordinating interactions between clathrin, accessory proteins, phosphoinositides and cargo proteins to ensure precise vesicle formation and internalization (Traub, 2009; Traub and Bonifacino, 2013; Azarnia Tehran et al., 2019). The µ2 subunit plays a crucial role in transmembrane cargo recognition through the YxxΦ motif (a tyrosine-based sorting signal) and in binding to the plasma membrane via two recognition sites for phosphatidylinositol-4,5-bisphosphate (PIP_2_) (Gaidarov and Keen, 1999; Jackson et al., 2010; Kovtun et al., 2020). This interaction drives a structural rearrangement of AP-2 from a ‘closed’ cytosolic state to an ‘open’ membrane-bound state, exposing the cargo-binding sites of both µ2 and σ2 (Jackson et al., 2010; Kovtun et al., 2020). Additionally, phosphorylation of Thr156 in the µ2 subunit further stabilizes the active conformation of AP-2 (Ricotta et al., 2002; Jackson et al., 2010).

The Arg170 residue, affected by the recurrent pathogenic variant, is located within a basic phospholipid-binding patch and is thought to play a crucial role in stabilizing membrane interactions and maintaining the open conformation of the protein (Jackson et al., 2010; Helbig et al., 2019). Structural analyses of the Arg170Trp variant result in thermodynamic instability of the AP-2 complex, particularly in its open conformation, potentially reducing cargo-binding efficiency. This was later confirmed through transferrin uptake assays (Helbig et al., 2019), demonstrating defects in endocytosis. Although the Arg170Trp variant in *AP2M1* appears to impair CME, the molecular mechanisms that give rise to the emergence of hyperexcitability and seizures remain unclear.

To investigate *AP2M1* dysfunction, we used *Drosophila melanogaster*, an established model organism for genetic epilepsies (Fischer et al., 2023). Previously, defects of individual AP-2 in *Drosophila* subunits have been shown to result in impaired CME (Choudhury et al., 2016). Flies with downregulated expression of individual AP-2 subunits, or mutations in σ2, exhibited alterations in neuromuscular-junction (NMJ) morphology, a phenotype frequently associated with CME defects (Koh et al., 2004; Verstreken et al., 2002; Stevens et al., 2012; Coyle et al., 2004). Furthermore, pan-neuronal RNA interference (RNAi) against the σ2 subunit resulted in heat-sensitive paralysis, a hallmark of CME dysfunction (Koh et al., 2004; Kosaka and Ikeda, 1983; Zinsmaier et al., 1994; Coyle et al., 2004). Interestingly, the temperature-sensitive dynamin mutant *shibire^ts^* can suppress seizures in flies with bang-sensitive mutations (Kroll et al., 2015). Similar seizure suppression was also observed with Rab GTPase mutations, altering endocytosis and suggesting a broader mechanism by which reduced CME activity may mitigate seizure susceptibility. Morphological investigations showed that pan-neuronal RNAi against any AP-2 subunit in L3 larvae caused altered NMJ bouton morphology (Choudhury et al., 2016). RNAi against the α-subunit of AP2 negatively impacted the development of class 4 dendritic arborization (c4da) neurons (Yang et al., 2011). A similar phenotype was observed for RNAi-mediated knockdown and loss-of-function mutations of *Nak*, which encodes numb-associated kinase and interacts with *AP-2µ* (Yang et al., 2011). The human ortholog of *Nak*, *AAK1*, phosphorylates *AP2M1* at Thr156, significantly enhancing the cargo-binding affinity of the µ2 subunit (Conner and Schmid, 2002; Ricotta et al., 2002). Reduced neuronal complexity was also observed in *shibire^ts^* larvae when reared at restrictive temperatures, reinforcing the link between CME dysfunction and impaired neuronal development (Yang et al., 2011).

To investigate the effects of *AP2M1* dysfunction, we employed RNAi-mediated knockdown of *AP-2µ* and generated a *Drosophila* model carrying the human p.Arg170Trp variant. We assessed the functional consequences through behavioral assays, imaging and electrophysiological seizure induction.

## RESULTS

### AP2M1 is highly conserved and the human pathogenic p.Arg170Trp variant disturbs a conserved phospholipid-binding patch

**Fig. 1.**
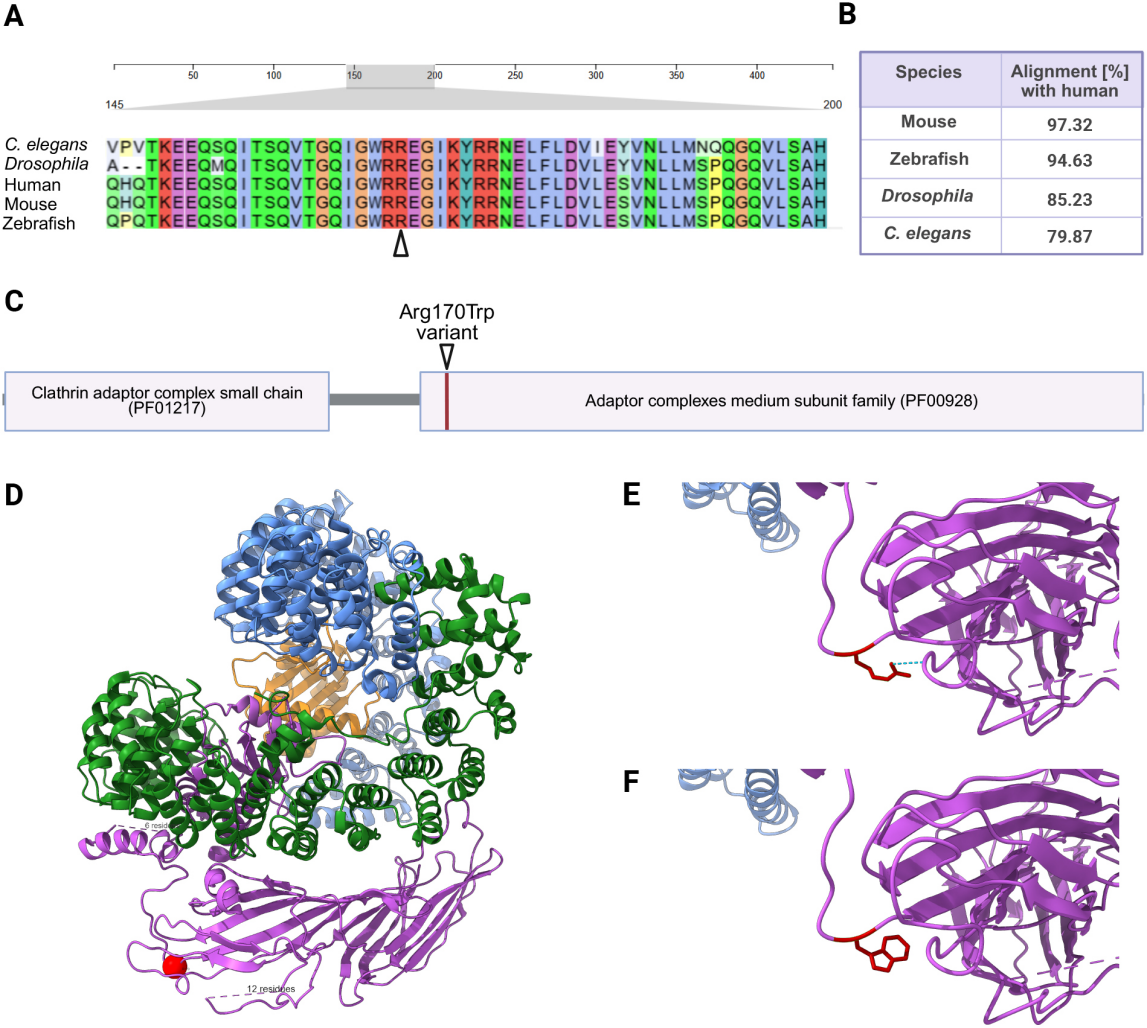
**Evolutionary conservation and protein domain structure of AP2M1.** (A) Clustal Omega Alignment (Madeira et al., 2024) of an AP2M1 segment bordering the Arg170 residue with ortholog genes from different species with Clustal2 color scheme. Red marks positively charged amino acids. The arrowhead marks the R170 position. (B) Pairwise alignment percentages of the AP2M1 protein between humans and other species. (C) Protein domains of AP2M1. The two major functional domains, the Clathrin adaptor complex small chain (PF01217) and the Adaptor complex medium subunit family (PF00928), are highlighted. The Arg170Trp variant is located in the N-terminal region of the μ homology domain, where it is part of a basic phospholipid binding patch. (D) Modeling of AP-2 in the membrane-bound form with ChimeraX 1.9 (Meng et al., 2023) based on Protein Data Bank (PDB): 6YAH (Kovtun et al., 2020). The R170W variant is located near the linker region of the µ2 subunit and is depicted as a red sphere. AP-2 subunits: AP-2α (blue), AP-2β (green), AP-2µ (magenta), AP-2σ (orange). (E) In the wild-type structure, the polar arginine residue at position 170 forms a hydrogen bond (blue dashed line) with the side chain of aspartate at position 428. (F) In the Arg170Trp variant, the polar arginine is replaced by a nonpolar tryptophan.

The adaptor related protein complex 2 subunit mu 1 gene (*AP2M1*) is evolutionarily highly conserved with a DIOPT score of 14/15 [DRSC (*Drosophila* RNAi Screening Center Integrative Ortholog Prediction Tool); Hu et al., 2011]. Alignment of the *Drosophila* AP-2µ sequence with *Caenorhabditis elegans*, zebrafish, mouse and human showed a high degree of overlap (Fig. 1A,B); 85.23% of AP-2µ aligns with the human AP2M1 protein sequence. The Arg170Trp variant is located in the N-terminal region of the C-terminal mu homology domain (PF00928) (Fig. 1C,D), which harbors the cargo-binding YxxΦ motif. The region neighboring Arg170 is largely identical across species and enriched with basic, positively charged amino acids (Fig. 1A). Notably, Arg170 is part of a conserved basic patch (residues Lys167, Arg169, Arg170, Lys421) that contributes to electrostatic interactions with negatively charged membrane components, such as phosphatidylinositol 4,5-bisphosphate (PIP₂), and is implicated in AP-2 membrane recruitment and cargo recognition (Jackson et al., 2010; Helbig et al., 2019). Replacement of arginine at the Arg170 residue with a large, hydrophobic amino acid such as tryptophan (Fig. 1E,F) might therefore negatively influence the cargo-binding capabilities of the AP-2 complex (Helbig et al., 2019; Kovtun et al., 2020).

### Modeling of AP2M1 dysfunction in *D. melanogaster*

**Fig. 2.**
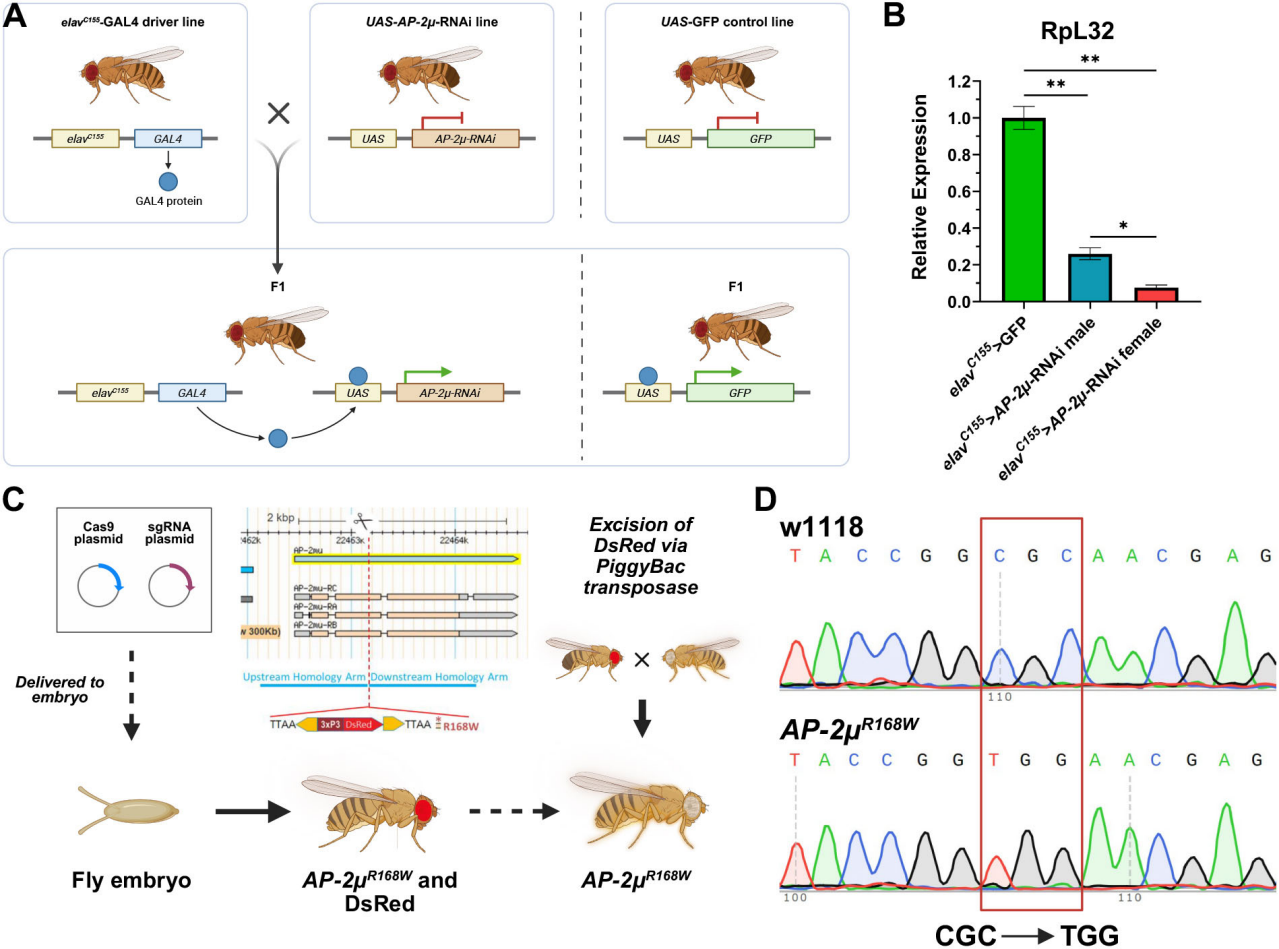
**Genetic approaches to model *AP-2µ* dysfunction.** (A) Crossing scheme to induce RNAi-mediated knockdown of *AP-2µ* with GFP expression as control. (B) Validation of *AP-2µ* knockdown by RT-qPCR. Expression levels of *AP-2µ* relative to *RpL32* show a significant reduction in *AP-2µ* -RNAi flies compared to controls. Nested *t*-test of six samples from two biological replicates of 20 fly heads each (*P<0.05, **P<0.01). (C) Schematic of the CRISPR-mediated mutagenesis workflow used to generate the *AP-2µ^R168W^* knock-in allele. The amino acid substitution R168W (homologous to human R170W) was introduced into the *Drosophila AP-2µ* locus using homology-directed repair. (D) Sanger sequencing chromatograms of the *AP-2µ* locus in w1118 and *AP-2µ^R168W^* flies. The red box highlights the nucleotide triplet coding for the amino acid at position 168, corresponding to a change from arginine to tryptophane in the protein.

To model *AP2M1* dysfunction in *Drosophila*, we used a knockdown and a humanization approach. First, we induced a pan-neuronal knockdown of *AP-2µ*, the *Drosophila* homolog of *AP2M1* by RNAi (Fig. 2A). Using the pan-neuronal *elav^C155^*-Gal4 driver line, we overexpressed a construct that produces double-stranded RNA targeting *AP-2µ* (Perkins et al., 2015). Expression of GFP via the *elav^C155^*-Gal4 driver line was utilized as control. The efficiency of the RNAi was confirmed by RT-qPCR of *AP-2µ* in adult fly heads (Fig. 2B). Expression of *AP-2µ* was reduced by approximately 74% in male flies and 93% in female flies. Second, we introduced the Arg168Trp variant (homologous to human Arg170Trp) into *AP-2µ* by CRISPR-mediated mutagenesis (Fig. 2C). Sanger sequencing confirmed successful genome editing at the target site, yielding homozygous viable mutant flies (Fig. 2D). Both the RNAi and humanized lines lacked spontaneous behavioral or morphological abnormalities and displayed normal development.

### Pan-neuronal RNAi against AP-2µ results in heat-sensitive paralysis

**Fig. 3.**
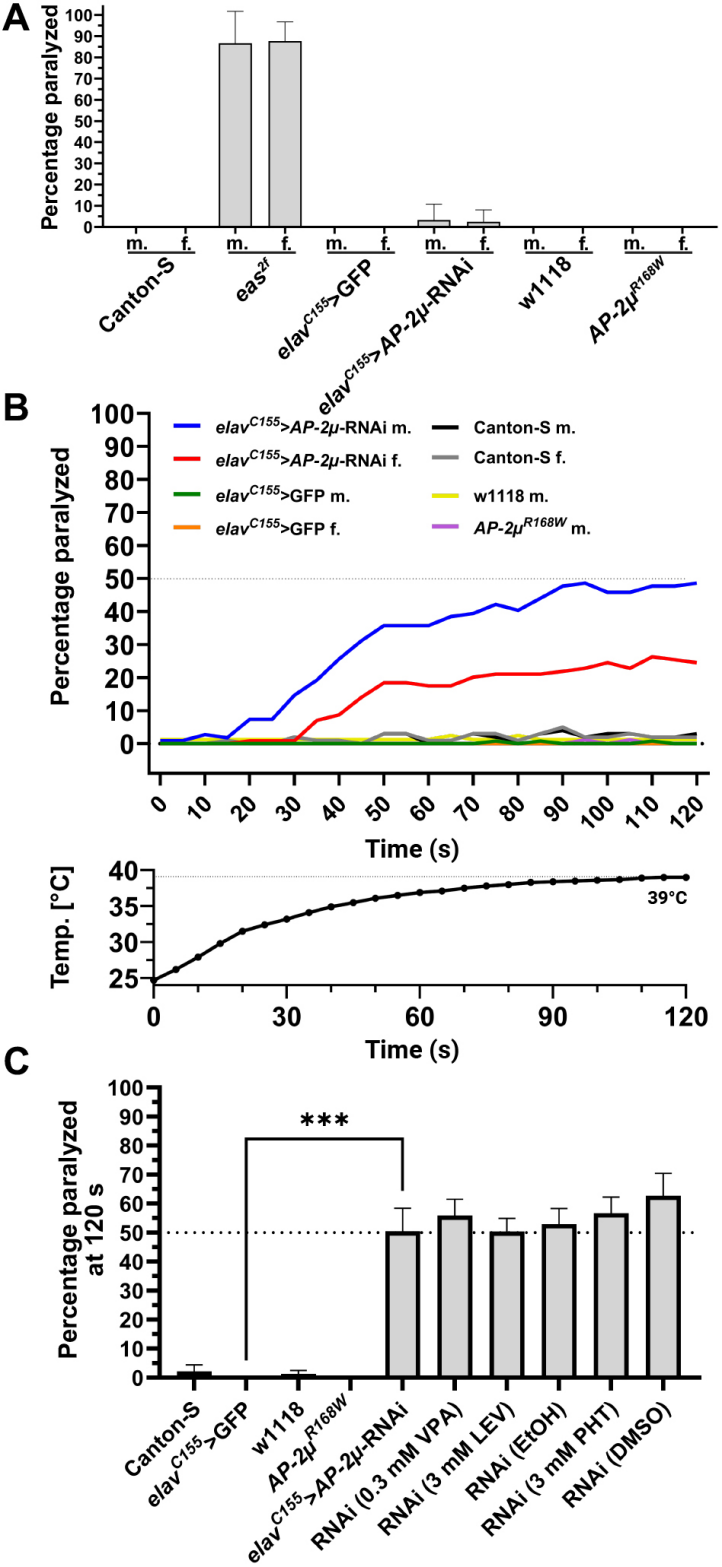
**Assessment of behavioral phenotypes using vortex and heat assays.** (A) Behavioral response to mechanical stimuli in the vortex assay, in which only bang-sensitive *eas^2f^* mutant flies showed paralysis (*n*>25 flies per condition). (B) Heat-induced paralysis of *AP-2µ*-RNAi flies in the heat assay. After 2 min in 40°C water, *elav^C155^-AP-2µ*-RNAi flies partially exhibit paralysis, unlike *elav^C155^-*GFP controls. Male flies were affected more strongly and nearly 50% were paralyzed at the end of the trial, while females exhibited a lower degree of paralysis (*n*>100 flies per condition, *n*=36 for *elav^C155^>GFP* female). w1118 and *AP-2µ^R168W^* male flies (*n*>80), as well as Canton-S flies (*n*=45) exhibited no heat-induced paralysis (*eas^2f^* and *nSyb*-*AP-2µ*-RNAi not shown). Air temperature in vials during the heat assay is shown below. The temperature rises steadily from ∼24°C in the beginning to 39°C in the end. (C) Percentage of flies paralyzed at the end of the heat assay after drug feeding for 2 days. Treatment of male RNAi flies with anti-seizure medication did not alleviate the paralysis (*n*>75 flies per treatment condition). EtOH, ethanol; LEV, levetiracetam; PHT, phenytoin; VPA, valproate. Unmarked, H_2_O. ***P<0.001 (Mann–Whitney *U*-test). f., female; m., male.

To test whether impairment of *AP-2µ* function caused seizure-like behavior in flies, we tested for bang sensitivity and heat sensitivity (Parker et al., 2011; Mituzaite et al., 2021; Fischer et al., 2024). *AP-2µ*-RNAi and *AP-2µ^R168W^* flies were tested alongside controls and the bang-sensitive *eas^2f^* flies. Whereas *eas^2f^* mutants (Kroll and Tanouye, 2013) displayed clear bang sensitivity in the vortex assay, neither *AP-2µ*-RNAi nor *AP-2µ^R168W^* flies were bang-sensitive (Fig. 3A). In the heat assay, *AP-2µ*-RNAi flies exhibited a pronounced paralysis phenotype (Fig. 3B). After 2 min of heat exposure, 48.62±5.51% (mean±s.d.) of the male and 24.56±3.96% of the female flies displayed paralysis, defined as a loss of posture with or without wing-buzzing. During the assay, the flies exhibited different behavioral states, shifting between paralysis and an upright position, with some flies remaining unaffected. Based on the observed behavior, we hypothesized that the phenotype might reflect seizure-like behavior, prompting us to test whether anti-seizure medications (ASMs) could alleviate it (Fischer et al., 2024). We applied 100 µl of dissolved ASMs onto the fly food (Fischer et al., 2024) and exposed flies to the food for 2 days. We used the three common ASMs, valproate (VPA), levetiracetam (LEV) and phenytoin (PHT), to treat AP-2µ-RNAi flies, using the respective solvents (water or ethanol) and DMSO as control. Drug uptake was previously validated using food coloring (Fischer et al., 2024). No significant change in the heat-sensitive phenotype was observed, with 50.33-62.66% of the flies remaining paralyzed by the end of the assay (Fig. 3C). *AP-2µ^R168W^* flies did not exhibit bang sensitivity or heat sensitivity. To test the impact of the rearing temperature on the phenotype known from other mutants (Garber et al., 2012), additional *AP-2µ^R168W^* flies were raised at 18°C, but this did not result in heat sensitivity. To assess whether one copy of the variant is sufficient to maintain *AP-2µ* function, we crossed the allele over a deficiency for *AP-2µ* (BDSC: 26537) (Cook et al., 2012), resulting in a fly line with only one functional copy of *AP-2µ* carrying the Arg168Trp variant. This cross did not lead to a heat-sensitive phenotype either. Further, RNAi against *AP-2µ* was induced via two different *nSyb*-Gal4 driver lines, resulting in no behavioral abnormalities in any assay.

Conclusively, pan-neuronal *AP-2µ*-RNAi in *Drosophila* induced a robust, sex-dependent, heat-sensitive paralysis phenotype that was unresponsive to common ASMs. Neither the *AP-2µ^R168W^* variant nor RNAi knockdown via an adult neuronal driver (*nSyb*-Gal4) produced comparable behavioral effects.

### *AP-2µ*-RNAi and *AP-2µ^R168W^* flies exhibit decreased susceptibility to electrically induced seizures

**Fig. 4.**
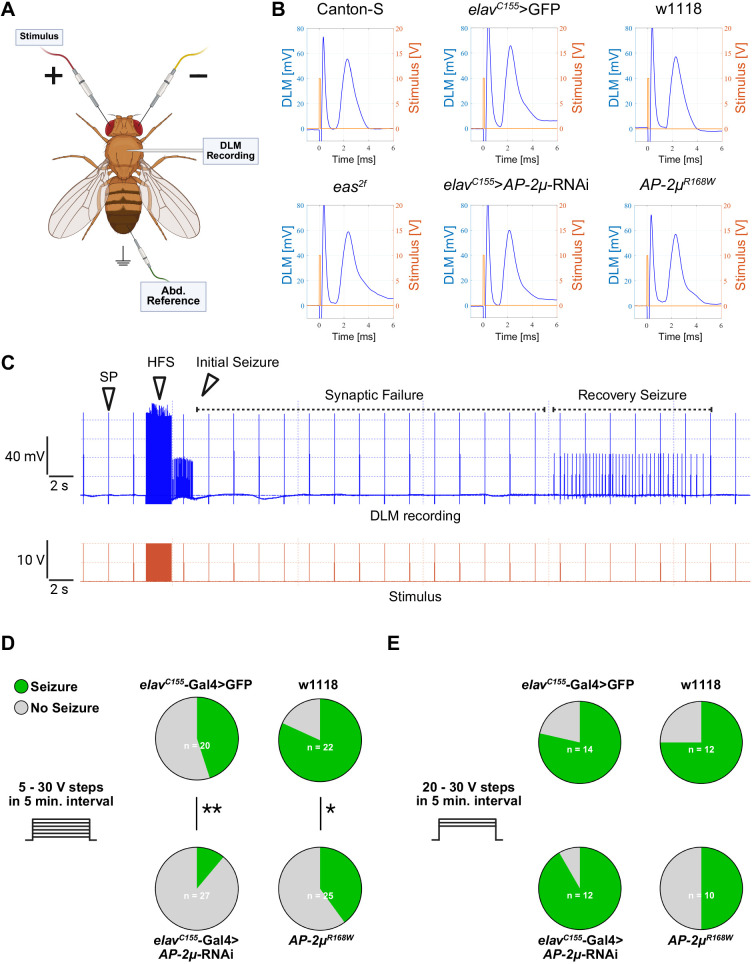
**Electrophysiological characterization of neuronal conductivity and seizure susceptibility of *AP-2µ*-RNAi and *AP-2µ^R168W^* flies.** (A) Electrophysiological setup including two tungsten stimulation electrodes in the brain, a saline-filled glass recording electrode in the DLM, and an abdominal tungsten electrode as reference. (B) Representative giant fiber response measurements at the DLM in response to a single pulse (SP) with 0.1 ms duration and 10 V amplitude at the brain with a latency of ∼1.4 ms. No differences were observed between *AP-2µ* deficiency, *eas^2f^* or control flies. (C) Representative electrophysiological recording from the DLM (blue trace, top) during stimulus application (orange trace, bottom) in a male Canton-S fly. A 2 s long HFS with 0.1 ms stimulus duration at 200 Hz induces seizure-like activity at the DLM. This is characterized by an initial seizure, a period of unresponsiveness of the GFS termed synaptic failure, and a recovery seizure, after which the GFS is responsive to 0.1 ms SPs again. (D) Incremental seizure induction protocol starting at 5 V HFS and increasing in 5 V steps every 5 min up to 30 V. Pie charts show the proportion of seizing flies at any time during the stimulation protocol. *AP-2µ*-RNAi and the *AP-2µ^R168W^* flies exhibited significantly less seizures. Fisher's exact test (*P<0.05, **P<0.01). The calculated voltage threshold for flies at which seizures could be induced is displayed in Table 1. (E) During the protocol starting at 20 V, no significant differences in seizure occurrence were observed.

To assess the impact of *AP-2µ*-RNAi or the R168W variant on neuronal conductivity, we performed electrophysiological recordings of the giant fiber system (GFS) at the dorsal longitudinal muscle (DLM) (Allen and Godenschwege, 2010) (Fig. 4A). When providing short, single pulses to the brain, no differences were observed in the GFS response to individual stimuli between the tested genotypes (Fig. 4B). To induce seizures, high-frequency stimuli (HFS) were delivered to the brain at different voltage levels (Kuebler and Tanouye, 2000; Lee and Wu, 2002; Saras et al., 2017). Successful seizure induction was characterized by aberrant high-frequency firing at the DLM, with distinct phases, including an initial seizure, synaptic failure and a recovery seizure (Fig. 4C). Canton-S flies were used to establish the seizure induction protocol, starting with a 5 V stimulus and increasing in 5 V increments every 5 min up to 30 V. In Canton-S flies, seizure induction succeeded consistently with an average seizure threshold of 11.54±1.04 V. For *eas^2f^* flies, the protocol was adapted to start at 1 V with incremental increases of 1 V to address the reduced seizure threshold. Seizures could be induced in all *eas^2f^* flies, with an average seizure threshold of 3.11±0.20 V (Table 1). When applying the 5 V step protocol in *AP-2µ*-RNAi and *AP-2µ*^R168W^ flies, we found that only a fraction of these flies responded to seizure induction (Fig. 4D). *AP-2µ*-RNAi flies exhibited a significantly lower rate of successful seizure inductions compared to the GFP-expressing controls (Table 1). Also, *AP-2µ*^R168W^ flies displayed significantly fewer seizures compared to controls (Fig. 4D, Table 1). However, the seizure induction threshold was similar between *AP-2µ*-RNAi and *AP-2µ*^R168W^ flies and their respective controls (Table 1), suggesting that other factors may contribute to the reduced likelihood of seizure occurrence. We next tested a second seizure induction protocol, starting at 20 V, to determine whether the incremental voltage increases in the primary protocol might be hampering seizure susceptibility. In the second protocol, we found no significant differences of seizure rate (Fig. 4E). This observation suggests a protective effect against seizures by sub-threshold stimulation in flies with *AP-2µ* dysfunction.

**Table 1.** Genotype and seizure threshold based on successful seizure inductions in the 5-30 V and 20-30 V experiments

Genotype	Seizure threshold (V)	Seizure induction in the 5 V protocol (5-30 V)	Seizure induction in the 20 V protocol (20-30 V)
Canton-S	11.54±1.04	100%	100%
eas^2f^ (1-6 V protocol)	3.11±0.20	100%	100%
*elav^C155^*>GFP	12.22±0.88	45%	78.57%
*elav^C155^*>*AP-2µ*-RNAi	11.67±1.67	11.11%	91.66%
w1118	13.33±0.81	81.82%	75%
*AP-2µ^R168W^*	16.00±0.67	40%	50%

Voltages are displayed as mean±s.e.m. Percentage of flies showing seizure-like activity at the DLM at any point during the protocol for 5-30 V and 20-30 V. Canton-S: 5-30 V *n*=13, 20-30 V *n*=13; *eas^2f^*: 1-6 V *n*=9, 20-30 V *n*=8.

In conclusion, *AP-2µ*-RNAi and *AP-2µ^R168W^* flies showed intact single-pulse GFS responses but a reduced proportion of HFS-evoked seizures under the incremental 5-30 V protocol, with unchanged thresholds among responders. Starting at 20 V, no difference in seizure rates could be observed, indicating an effect of the incremental protocol on seizure susceptibility in flies with *AP-2µ* dysfunction.

### *AP-2µ*-RNAi flies exhibit altered morphology of c4da neurons

Dysfunction of endo- and exocytosis has been linked to abnormal neuron morphology in *Drosophila* (Yang et al., 2011; Choudhury et al., 2016; Peng et al., 2015; Zong et al., 2018). To investigate the role of *AP-2µ* in controlling neuron morphology, we investigated the effects of *AP-2µ*-RNAi at the NMJ and in c4da neurons.

**Fig. 5.**
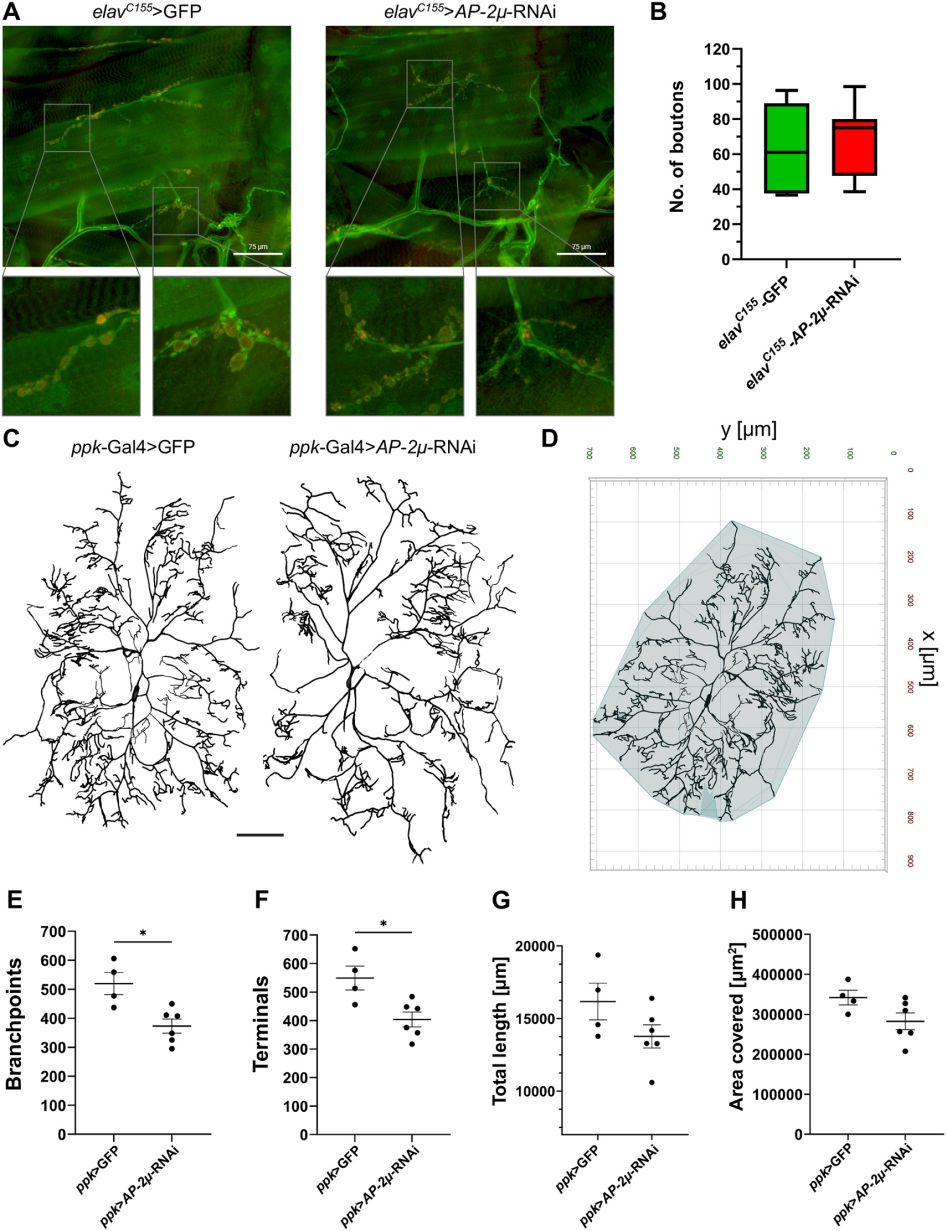
**Morphological and synaptic defects upon *AP-2µ*-RNAi.** (A) Representative images of NMJs in third instar larvae expressing either *elav*-GFP (left) or *elav*-*AP-2μ*-RNAi (right), stained with anti-HRP (green) and anti-Csp (red). Magnified views of individual boutons are shown beneath. NMJ morphology was heterogenous within genotypes, but no consistent or overt differences were observed between RNAi and control animals. (B) Analysis of bouton number per image. Approximately three images of NMJ morphology were analyzed per animal, *n*=7 per group. No significant differences were found using Mann–Whitney *U*-test. Whiskers represent minimum/maximum, horizontal line the median, and box limits the 25th and 75th percentiles. (C) Representative reconstructions of c4da neurons in L3 larvae expressing either *ppk*-Gal4>GFP as control (left) or *ppk*-Gal4>*AP-2µ*-RNAi (right). Neurons are oriented with anterior to the left and lateral to the top of the image. The *AP-2µ*-RNAi-expressing neurons show reduced branching complexity compared to controls. Scale bar: 100 µm. (D) A custom pipeline and Python script were used in arivis Vision4D to calculate the smallest convex polygon enclosing all dendritic branches, providing a quantitative measure of the neuron's spatial extent. (E,F) RNAi against *AP-2µ* led to a reduction in number of branch points and end terminals. (G,H) The total length of processes and the area covered by the dendritic arbor did not change significantly. Values displayed with s.e.m.; Mann–Whitney *U*-test (*P<0.05).

At the larval NMJ, overall bouton morphology was largely preserved in the *AP-2µ*-RNAi compared with GFP controls. Boutons in RNAi larvae occasionally appeared smaller or more numerous, but this was inconsistent and seen in some controls as well. Quantification across approximately three fields per larva (*n*=7 per genotype) revealed no significant difference in bouton number (controls: 62.57±9.47; RNAi: 69.81±7.67 boutons per image) (Fig. 5A,B).

In c4da neurons, we found that *AP-2µ*-RNAi led to a reduction in branching pattern complexity (Fig. 5C). Specifically, the number of branch points and terminals were reduced (branch points: control 519.8±38.36, *AP-2µ*-RNAi 371.8±24.23; terminals: control 549.3±42.11, *AP-2µ*-RNAi 403.8±26.08) (Fig. 5E,F), but the total length of processes showed no difference (control 16,174±1264 µm; *AP-2µ*-RNAi 13,772±794.5 µm) (Fig. 5G).

Convex hull analysis (Fig. 5D) further revealed that the maximum spatial extent of the neurons remained unchanged upon *AP-2µ*-RNAi (control 341,955±18,200 µm^2^, *AP-2µ*-RNAi 282,582±21,074 µm^2^) (Fig. 5H). Taken together, the RNAi against *AP-2µ* in c4da neurons reduced the complexity of dendritic arborization. In conclusion, *AP-2µ*-RNAi left NMJ bouton number and gross morphology largely unchanged but reduced dendritic branching complexity in c4da neurons. Both branch points and terminals were decreased in number, but total arbor length and spatial extent remained unaffected.

## DISCUSSION

In this study, we used *D. melanogaster* to model *AP2M1-*associated DEE by generating a *Drosophila* line with pan-neuronal knockdown by RNAi of *AP-2µ*, the *Drosophila* ortholog of the human *AP2M1* gene, as well as a CRISPR/Cas9-engineered humanized fly carrying a Arg168Trp variant, analogous to the recurrent human Arg170Trp variant (Helbig et al., 2019). In summary, *AP-2µ*-RNAi flies exhibited a stable phenotype, characterized by thermosensitivity and alterations of neuronal morphology. Specifically, the heat-induced paralysis observed in these flies was reminiscent of heat-induced seizures observed in flies carrying pathogenic variants in the *SCN1A*-analogous fly gene *para* (*para^GEFS+^* or *para^DS^*), which mimic *SCN1A*-associated epilepsy syndromes (Sun et al., 2012; Schutte et al., 2014; Roemmich et al., 2021). Whereas *para* flies remain paralyzed throughout the trial, the *AP-2µ-*RNAi flies shifted between seizure-like behavior and upright positions, which was indicative of a non-seizure origin of the paralysis. Furthermore, the paralysis phenotype upon *AP-2µ*-RNAi was not responsive to anti-seizure treatment (Fischer et al., 2024) and flies exhibited a deceased susceptibility to electrical seizure induction, contrary to the initial hypothesis. Interestingly, male flies were affected twice as much by heat-induced paralysis than female flies. Surprisingly, the knockdown was not stronger in male flies, even though the expression of *elav*-Gal4 is X-linked (Belyi et al., 2020). This could point to other sex-specific differences, such as hormonal or metabolic differences between male and female flies (Dahn and Wagner, 2025). *AP-2µ^R168W^* flies showed a similar, albeit less pronounced, reduction in seizures. These findings underline that *AP-2µ* deficiency in *Drosophila* is associated with a reproducible phenotype that is, however, not concordant with the epilepsy phenotype observed in humans. Phenotype discrepancies are also commonly found for fly models of other genetic disorders (Yamamoto et al., 2014; Bellen et al., 2019). For instance, the *para^ts1^* allele also shows heat sensitivity along with increased resistance to seizure induction, a similar phenomenon to what we observed in the *AP-2µ*-RNAi flies in the present study (Grigliatti et al., 1973; Pavlidis and Tanouye, 1995).

Previous clinical and functional data from our group suggest that the Arg170Trp variant causes impairment of CME by destabilizing the AP-2 complex, leading to defective synaptic vesicle recycling and a reduction in transferrin uptake in cell culture assays (Helbig et al., 2019). Additionally, a heat-sensitive paralysis phenotype has been described for the knockdown of *AP-2σ* in *Drosophila*, which encodes another AP-2 subunit (Choudhury et al., 2016). Mutations such as *shibire^ts^* also show thermosensitive paralysis (Kosaka and Ikeda, 1983). Interestingly, whereas knockdown of the α and β2 subunits of AP2 was found to be lethal, knockdown of the µ2 subunit did not result in any observable phenotype (Choudhury et al., 2016), which contrasts with our findings. This discrepancy might be attributed to accumulated genetic differences between strains maintained in different laboratories or differences in the methodologies used for heat assays, such as using a sushi cooker for heating (Choudhury et al., 2016).

Given the resistance to anti-seizure treatment and electrical seizure induction, we hypothesize that the heat sensitivity in *AP-2µ*-deficient flies is similar to defects observed in other CME genes and likely associated with a failure in synaptic transmission. This could be caused by insufficient replenishment of synaptic vesicles (Choudhury et al., 2016; Jung et al., 2015; Rohrbough and Broadie, 2002). Of note, the s*hibire^ts^* allele – *shibire* being orthologous to human *DNM1* – also causes heat-related paralysis (Kosaka and Ikeda, 1983). Dynamin, the gene product of *shibire*, is a membrane-remodeling GTPase crucial for membrane fission, the final step in CME (Ferguson and De Camilli, 2012). Dynamin deficiency severely impairs synaptic vesicle recycling and leads to altered membrane structures with an accumulation of collared pits (Kosaka and Ikeda, 1983). Interestingly, *shibire^ts^* also reduces seizure occurrence in established *Drosophila* seizure models, including those involving *para*, *eas* or *sda* (also known as *jus*) (Kroll et al., 2015). In these flies, heat treatment significantly reduced seizure susceptibility in behavioral and seizure-induction assays. This effect was not limited to *shibire^ts^* but was also observed in mutants of Rab GTPases, which regulate vesicle trafficking (Kroll et al., 2015). Consequently, it was proposed that altering or reducing vesicle recycling might serve as a seizure-suppressing mechanism and could be a potential therapeutic target for epilepsies. It is challenging to pinpoint such paralysis as either a result of seizure-like activity or disturbed neuronal signaling, as spontaneous discharges of the DLM have been observed in *shibire^ts^* and could reflect impaired inhibitory control due to disrupted vesicle recycling, which might play a role in seizure generation as well (Kroll et al., 2015).

In line with our findings, the human dynamin gene *DNM1* is associated with another form of DEE (von Spiczak et al., 2017), supporting the idea of differing phenotypical outcomes of CME abnormalities across species. One possible explanation for a reduction in electrically induced seizures in CME deficient lines could be the reduced supply of recycled vesicles at the presynaptic membrane. Our observation that electrical seizure induction starting at 5 V resulted in a lower overall likelihood of seizure occurrence, compared to the protocol starting at 20 V, suggests that repetitive sub-seizure threshold stimulation could lead to cumulative vesicle depletion. This is corroborated by studies in *AP-2σ* mutants, in which impaired vesicle regeneration during high-frequency stimulation at the NMJ leads to a progressive decline in synaptic transmission and neurotransmitter release (Choudhury et al., 2016).

This raises the question of how seizures can be explained in humans carrying variants in *AP2M1*, *DNM1* and other genes involved in CME, such as *CDKL5* (Kontaxi et al., 2023), *CLTC* (Sveistrup and Myers, 2024) or endophilin (Milosevic et al., 2011). A plausible explanation could be that CME defects do not directly cause neuronal hyperexcitability through alterations in synaptic transmission, but rather more generally by affecting neuronal development. Indeed, CME defects have been found to alter neuronal morphology in developing flies. In particular, morphological and functional alterations of NMJ boutons have been observed in *Drosophila* lines with RNAi against other AP-2 subunits (Choudhury et al., 2016). Similar effects have also been observed in defects of other CME genes, such as *shibire* (Dickman et al., 2006), *EndoA* (Guichet et al., 2002; Dickman et al., 2006; Rikhy et al., 2002), *Dap160* (Koh et al., 2004), *Eps15* (Koh et al., 2007), *stnA* (Petrovich et al., 1993; Stimson et al., 1998), *stnB* (Mohrmann et al., 2008), *nwk* (Coyle et al., 2004), *Rab11* (Khodosh et al., 2006; Liu et al., 2014), and several others. Although we expected altered NMJ morphology, we could not reliably observe this phenotype and focused on a different class of neurons.

When monitoring neuronal morphology in c4da neurons, we found that *AP-2µ*-RNAi larvae exhibited less-complex dendrite arborization patterns. This is consistent with previous studies in *shibire^ts^* and *AP-2α* knockdown flies, which also exhibited reduced dendritic complexity in c4da neurons (Yang et al., 2011; Peng et al., 2015). Similarly, flies carrying the *AP-2µ^NN20^* loss-of-function allele show defects in dendrite pruning (Zong et al., 2018). Furthermore, c4da-neuron morphology is also disrupted in other CME deficiency lines, such as *Rab5* (Takayama et al., 2014; Satoh et al., 2008; Copf, 2014), *Nak* (Yang et al., 2011) and *Dab* (Hattori et al., 2013).

Further investigation into the phenotypic differences between flies and humans could provide valuable insights into *AP2M1*-DEE. Over the past decade, our understanding of DEE has evolved. Traditionally, epileptic activity itself was considered the primary cause of cognitive and behavioral impairment (Berg et al., 2010), leading to the now less frequently used term ‘epileptic encephalopathy’. While this concept may apply to a subset of disorders, it has become increasingly clear that many DEE-associated conditions are associated with developmental deficits that occur independently of seizure activity, remain unaffected by seizure treatments, and, in some cases, even precede the onset of seizures (Scheffer et al., 2017). For instance, in Dravet syndrome, developmental regression and cognitive decline often arise prior to significant EEG abnormalities (Connolly, 2016). To reflect these findings, the International League against Epilepsy coined the term ‘developmental and epileptic encephalopathy’ to acknowledge both developmental deficits and seizure activity as separate but interconnected aspects of these disorders.

In the case of *AP2M1*-DEE, our findings suggest that the developmental consequences of *AP2M1* loss of function may underlie the more substantial burden of the human disease phenotype, with epilepsy possibly being a secondary phenomenon resulting from defects of neuronal development. This aligns with clinical observations where some of the reported patients, despite achieving seizure freedom, did not experience improvements in cognitive and behavioral outcomes (Helbig et al., 2019). Furthermore, the co-occurrence of epilepsy with ataxia and muscular hypotonia points to a broader impact on the nervous system beyond cortical neurons.

Previous studies in *Drosophila* have demonstrated that disrupting neuronal activity during critical development windows can lead to persistent seizure-like behavior in adult flies (Giachello and Baines, 2015; Hunter et al., 2024), without the presence of a seizure mutation. Similarly, in mice, interference with neuronal activity during critical periods of neurogenesis exacerbates seizure phenotypes (Lybrand et al., 2021). Future studies should aim to identify critical time windows for developmental *AP2M1* deficiency in *Drosophila* and mammalian models and explore whether targeted interventions during these periods can mitigate disease severity.

### Conclusion

In this study, we investigated *AP2M1* dysfunction in *Drosophila* through *AP-2µ*-RNAi and a CRISPR/Cas9-engineered *AP-2µ^R168W^* variant. Whereas RNAi led to heat-induced paralysis and neurodevelopmental defects, both models showed decreased susceptibility to electrically induced seizures. This aligns with findings that CME dysfunction can suppress seizures in flies, suggesting that disruptions in vesicle recycling affect neuronal excitability differently across species. Our results underscore the role of CME in neurodevelopment and synaptic function, highlighting the need to investigate how endocytic defects contribute to epilepsy in humans and whether modulating vesicle trafficking could offer therapeutic potential.

## MATERIALS AND METHODS

### Fly husbandry and utilized stocks

All *D. melanogaster* flies were maintained on standard cornmeal food at 25°C with a 12-h light/dark cycle. Fly lines were obtained from the Bloomington *Drosophila* stock center (BDSC), from other laboratories as indicated, or were specifically created. The following fly lines were used in this study: Canton-S (BDSC: 64349), *P{w[+mW.hs]=GawB}elav[C155]* (*elav-*Gal4) (BDSC: 458), *y[1] v[1]; P{y[+t7.7] v[+t1.8]=TRiP.JF02875}attP2/ TM3, Sb[1]* (*AP-2µ-*RNAi) (BDSC: 28040), *P{y[+t7.7] v[+t1.8]=TRiP.JF02875}attP2/ TM3, P{w[+mC]=ActGFP}JMR2, Ser[1]* (*AP-2µ-*RNAi) (BDSC: 28040 with balancer from 4534), *y[1] v[1]; P{y[+t7.7] v[+t1.8]=UAS-GFP.VALIUM10}attP2* (*UAS-*GFP) (BDSC: 35786), *[LWG228] w[1118]* (WellGenetics Inc., Taiwan), w[*];; AP-2µ[R168W]/(TM3, Sb[1]) (*AP-2µ^R168W^*) (WellGenetics Inc., with *TM6B, Tb[1]* as original balancer), *w[1118]; Df(3R)BSC685/TM6C, Sb[1] cu[1]* (*AP-2µ* Df) (BDSC: 26537), *w, eas^2f^* (*eas*) (Richard Baines, University of Manchester, UK), *UAS-dcr2/ CKG; UAS-mcD8-GFP, ppk-Gal4* (*ppk*-Gal4) (Gaia Tavosanis, RWTH Aachen University, Germany), *y[1] w[1118]; P{y[+t7.7] w[+mC]=nSyb-GAL4.P}attP2* (*nSyb-*Gal4) (BDSC: 51941), y[1] w[*]; P{w[+m*]=nSyb-GAL4.S}3 (*nSyb-*Gal4) (BDSC: 51635).

### Introduction of the human p.Arg170Trp variant into the fly genome via CRISPR/Cas9

The amino acid position of the human p.Arg170Trp (R170W) variant is analogous to position 168 in the *Drosophila* AP-2μ protein based on protein sequence alignment via Clustal Omega Multiple Sequence Alignment (EMBL-EBI). To create a humanized fly gene, CRISPR-mediated mutagenesis was carried out by WellGenetics Inc. (Taiwan) using a modified homology-dependent repair strategy (Kondo and Ueda, 2013). Guide RNA (gRNA) sequences targeting *AP-2µ*, TCACGTACTCCAATACGTCC[AGG] and GACCCTGCGGGCTCATCAGC[AGG], were cloned into U6-promoter plasmids. A donor plasmid was constructed in a pUC57-Kan backbone, which included two homology arms: the R168W codon change (CGC→TGG) and a 3xP3-DsRed marker cassette flanked by PiggyBac terminal repeats. Together with hs-Cas9 and the gRNA plasmids, the donor was injected into w[1118] embryos. Homology-dependent repair resulted in insertion of the DsRed marker into exon 2 of *AP-2µ*, and positive transformants were selected by eye-specific fluorescence. To remove the selection marker, flies were crossed to a source of PiggyBac transposase. Excision left behind a single TTAA motif embedded in the coding exon along with the R168W point mutation and a silent codon change at position I164 (I164I). Sequencing confirmed the incorporation of the R168W variant and successful excision of the *PBacDsRed* marker in line 220862ex2. The resulting line termed *AP-2m^R168W^* was balanced over TM6B, Tb[1] and later TM3 and Sb[1]. Although the DsRed-marked allele was only viable in heterozygous flies, homozygous viability was restored after marker removal. The unaltered w[1118] strain acted as control.

### *AP-2µ*-RNAi and quantification of knockdown efficiency by RT-qPCR

To model loss-of-function effects of *AP-2µ*, we first induced pan-neuronal RNAi by expressing a double-stranded RNA against *AP-2µ* mRNA [Transgenic RNAi Project (TRiP) line: BDSC 28040] under the control of the pan-neuronal driver *elav^C155^*. Control groups included Canton-S and flies expressing *P{y[+t7.7] v[+t1.8]=UAS-GFP.VALIUM10}attP2* with the *elav*-Gal4 driver, which is also used for the RNAi, following BDSC TRiP recommendations for RNAi experiments. Adult fly heads (3-5 days post-eclosion) were collected, and total RNA was extracted using a TRIzol-based protocol. Briefly, 20 frozen fly heads per sample were homogenized in TRIzol (Invitrogen) with 1.2-1.4 mm ceramic beads (Mühlmeier) using a SpeedMill (Analytik Jena). After chloroform addition and centrifugation (12,000 ***g***, 15 min, 4°C), the aqueous phase was collected, RNA was precipitated with isopropanol, washed with 75% ethanol, air-dried, and resuspended in nuclease-free water (QIAGEN). RNA concentrations were measured using a NanoDrop ND-100 spectrophotometer (VWR International) and sample concentrations were normalized to the lowest concentration among the samples. cDNA synthesis was performed by reverse transcription, followed by qPCR using gene-specific primers for *AP-2µ* and normalized against the housekeeping gene *RpL32*. For this, 15 µl RNA per sample were collected and an iScript cDNA Synthesis Kit (Bio-Rad Laboratories) was used to generate cDNA. To each RNA sample, 4 µl of reaction mix and 1 µl of reverse transcriptase enzyme were added, followed by the addition of nuclease-free water to adjust the total reaction volume to 20 µl. The cDNA synthesis reaction was performed using a T-Professional Basic thermocycler (Biometra) with the following conditions: priming at 25°C for 5 min, reverse transcription at 46°C for 20 min and a final enzyme inactivation step at 95°C for 1 min. For qPCR, the generated cDNA was quantified using a QuantStudio I apparatus (Applied Biosystems). Samples were prepared with 1× iQ SYBR Green Supermix (Bio-Rad Laboratories), 5 pmol of each oligonucleotide primer and 2.5-fold diluted synthesized cDNA for a total volume of 20 µl and pipetted as triplicates on a 384-well plate (Bio-Rad Laboratories). The samples were then run for 3 min at 95°C, 40 cycles of 15 s at 95°C and 1 min at 60°C. For *AP-2µ*, the forward primer 5′-TCTTCCACATCAAGAGAGCAAA-3′ and reverse primer 5′-GCCGAAGTAGGATTGCATCAC-3′ were used; for *RpL32*, the forward primer 5′-TGCTAAGCTGTCGCACAAATG-3′ and reverse primer 5′-ATCCGTAACCGATGTTGGGC-3′ were used. Gene expression levels were quantified using the ΔΔCt method (Livak and Schmittgen, 2001), comparing *AP-2µ* transcript levels in *elav-AP-2µ*-RNAi flies to the controls. Off-target effects are not reported for the utilized RNAi line based on UP-TORR (Hu et al., 2013), but remain a possibility. Statistical significance was assessed using an unpaired *t*-test.

### Drug feeding and experimental preparation

For behavioral testing, adult flies aged 1-3 days post-eclosion were collected into food vials using CO₂ anesthesia (maximum 15 animals per vial). The vials were prepared with 100 µl of dissolved ASMs, which were pipetted onto the food and left to completely dry. The following concentrations and compounds were used: 0.3 mM VPA (Sigma-Aldrich) in water, 3 mM LEV (Sigma-Aldrich) in water, 3 mM PHT (Sigma-Aldrich) in ethanol, based on previous publications (Fischer et al., 2024; Pandey and Nichols, 2011). Solvents were used as controls. Flies were left on the prepared food for 2 days and used for experiments 3-5 days post-eclosion. To ensure comparability, flies in all experiments received solvent (water)-treated food.

### Behavioral assays

For behavioral testing, a vortex assay and a heat assay were employed. Flies were collected into empty vials using ice anesthesia with ten animals per vial for the vortex assay or five animals per vial using CO₂ anesthesia for the heat assay. Animals were left to recover for a minimum of 1 h. For the vortex assay, flies were subjected to mechanical stress using a vortex mixer (Vortex Genie 2m, Scientific Industries) at maximum speed for 10 s (Kuebler and Tanouye, 2000; Mituzaite et al., 2021; Fischer et al., 2024). Behavior was video recorded and seizure probability (ratio of seizing to non-seizing flies) was determined. For the heat assay, vials containing flies were placed in a water bath at 40-41°C for 120 s. Fly behavior was video recorded and analyzed in 5 s intervals. To evaluate paralysis, all flies that lost posture or showed wing buzzing were counted as paralyzed.

### Electrophysiology of the *Drosophila* nervous system

To determine the seizure susceptibility of the flies and functionality of the GFS, *in vivo* electrophysiological recordings at the DLM were performed (Kuebler and Tanouye, 2000; Lee and Wu, 2002; Allen and Godenschwege, 2010). Flies were mounted on non-poisonous glue traps (Gelbtafeln; Inseko) for recordings. A tungsten electrode serving as ground electrode was inserted in the abdomen of the fly and a saline-filled glass electrode of ∼10 MΩ resistance (Allen and Godenschwege, 2010) was inserted in the DLM 45a (Allen and Godenschwege, 2010; Gu and O'Dowd, 2006). Two tungsten electrodes were inserted in the brain for stimulation. Signals from the DLM were recorded using an EXT-10-2F extracellular amplifier, housed in an EPMS-07 (npi Electronic GmbH). Signals were digitalized via a CW-INT-20USB interface (npi Electronic GmbH) and recorded in WinEDR software (University of Strathclyde, Glasgow, UK). Analysis was performed in WinEDR or MATLAB using a custom script (The Mathworks Inc.). An ISO-01M-100 stimulus isolator (npi Electronic GmbH) was used to deliver electric stimuli to the brain. Activation of the GFS was achieved by monophasic 10 V stimuli with 0.1 ms duration. Short single pulses with 0.1 ms duration and 10 V amplitude were used to activate the GFS, leading to a voltage response at the DLM after approximately 1.4 ms. Seizures were induced using an HFS of varying voltage with 0.1 ms duration, 200 Hz frequency and 2000 ms total stimulus length. Two types of seizure induction protocols were used. The first protocol aimed to determine the seizure threshold of the tested flies starting at 5 V HFS with increments of 5 V every 5 min until 30 V. The second protocol started at 20 V with 5 V increments every 5 min until 30 V. During both protocols, giant fiber functionality was monitored by a 0.1 ms pulse with 10 V amplitude every 2 s. Successful seizure induction manifested in the DLM as characteristic seizure-like activity (Lee and Wu, 2002).

Metal electrodes were fabricated from 0.1 mm tungsten wire (Hürkey et al., 2023), which was electrochemically sharpened in a 1 M KOH solution. The tungsten wire was attached to the anode of the stimulus isolator, while a platinum wire was attached to the cathode. The platinum wire was placed inside the KOH solution and a monophasic current (100 Hz, 40 V and 1 ms duration) was applied. The tungsten wire was repeatedly lowered into the solution until a conic-shaped tip formed. Afterwards, it was rinsed with deionized water.

### Imaging of c4da neurons in L3 larvae

To evaluate the morphology of c4da neurons in flies with *AP-2µ* dysfunction, the gene was knocked down by RNAi specifically in c4da neurons using a *ppk*-Gal4 driver line, with knockdown strength enhanced by co-expression of *Dicer-2* (Kim et al., 2006). Images were acquired using a Zeiss LSM900 confocal microscope (Carl Zeiss AG). Larvae in the L3 stadium were collected and washed. Only larvae showing GFP expression exclusively in c4da neurons were selected for imaging, as GFP in other tissues indicated the presence of a balancer chromosome. A single larva was then placed onto a microscope slide with a drop of halocarbon oil 27. A glass coverslip was used to flatten out the larva and fixed with double-sided tape. Neurons of the abdominal hemi-segment 4 were imaged and reconstructed using arivis Vision4D software (Carl Zeiss AG). In the reconstructed neurons, the number of branch points, number of terminals and total dendritic length were analyzed and compared. Branch points were defined as sections in the dendrite where a single process bifurcates into two or more branches; terminals were identified as the endpoints of dendritic branches, representing the final points of neuronal outgrowth. The spatial extent of the neurons was quantified using a convex hull analysis, which assesses the outer boundary enclosing all dendritic branches. Following 3D reconstruction, a pipeline-generated object of the dendritic arbor was used to compute the minimal convex volume enclosing all branches, using a custom script provided by arivis (ZEISS).

### Imaging of the NMJ in L3 larvae

The NMJ of L3 larvae was imaged using an Echo Revolution automated hybrid widefield microscope (Echo Inc.). The larvae were dissected in cold PBS and the fillet stretched out using pin needles (Brent et al., 2009). Larvae were then fixed for 20 min in 4% paraformaldehyde and washed with PBS containing 0.3% Triton X-100 (PBS-T). Then, the samples were blocked with 10% goat serum in PBS. Primary antibody staining was performed with mouse anti-CSP (1:50) [DCSP-3 (1G12), Developmental Studies Hybridoma Bank] and rabbit anti-HRP (1:100) (500-8634-HRP, AbboMax) antibodies in blocking solution overnight at 4°C. Samples were washed again with PBS-T and secondary antibody incubation was conducted with goat anti-mouse Alexa Fluor 555 and goat anti-rabbit Alexa Fluor 488 (1:750) (A-48287 and A-32731, Invitrogen, Thermo Fisher Scientific) antibodies for 3-5 h at room temperature. Samples were then mounted in Vectashield antifade mounting medium without DAPI (H-1000-10, Vector Laboratories) and sealed with clear nail polish. Imaging was accomplished with an ECHO Revoultion automated hybrid widefield epifluorescence microscope with 20× and 40× air objectives and ECHO's integrated software.
